# Supplementary figures and images for: Spatial clustering of CD68+ tumor associated macrophages with tumor cells is associated with worse overall survival in metastatic clear cell renal cell carcinoma
Source: PLoS One. 2021 Apr 21;16(4):e0245415. doi: 10.1371/journal.pone.0245415 (PMC8059840; doi:10.1371/journal.pone.0245415)

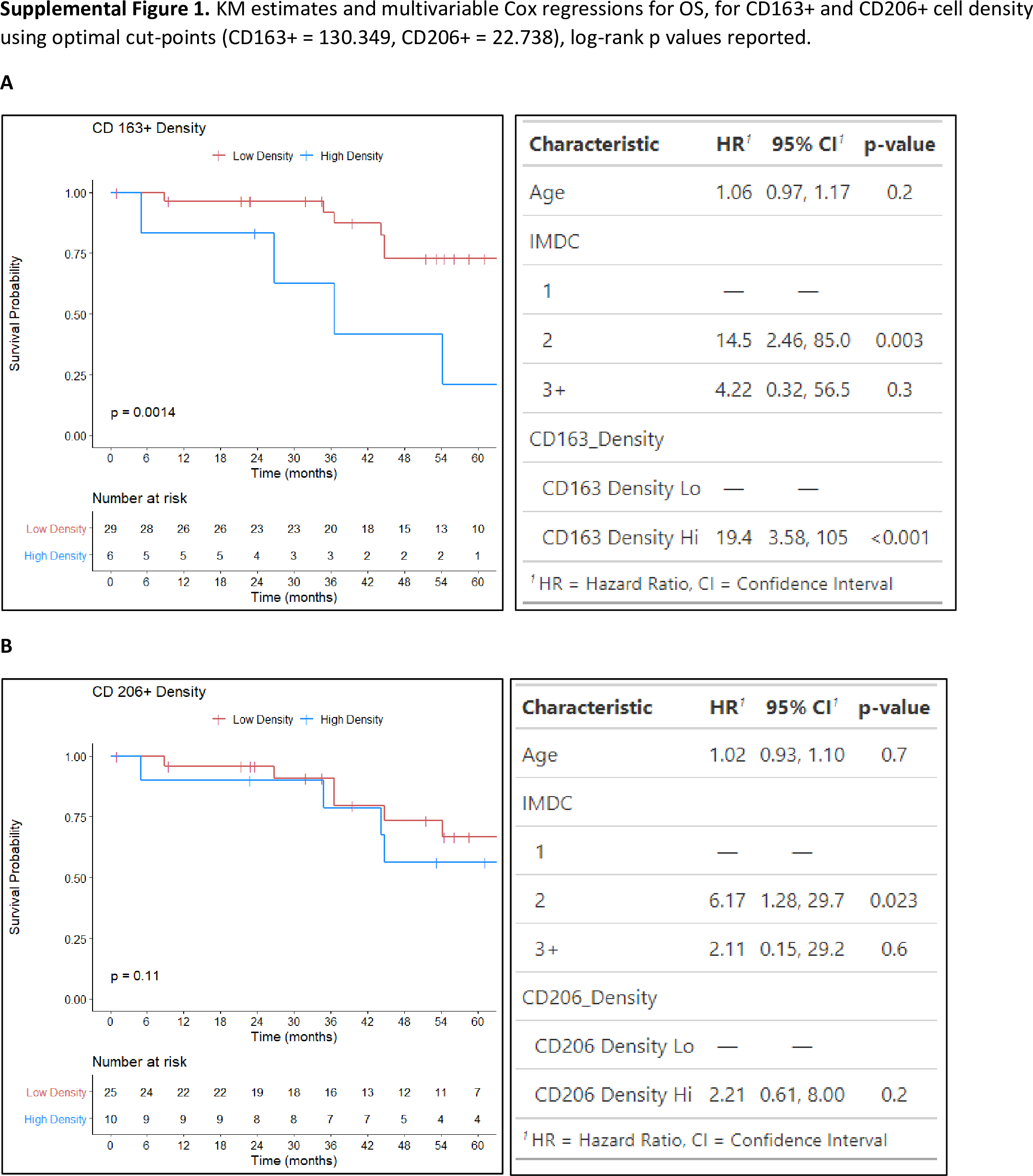

Supplement: S1 Fig — (TIF) [file pone.0245415.s001.tif]
