# Supplementary material for: Spatial clustering of CD68+ tumor associated macrophages with tumor cells is associated with worse overall survival in metastatic clear cell renal cell carcinoma
Source: PLoS One. 2021 Apr 21;16(4):e0245415. doi: 10.1371/journal.pone.0245415 (PMC8059840; doi:10.1371/journal.pone.0245415)
Supplement: Attachment — Submitted filename: Response to reviewers.docx [file pone.0245415.s003.docx]

**RESPONSE TO REVIEWERS**

We would like to thank the reviewers for their careful reading of the manuscript and thoughtful feedback. Our responses are below in RED.

Reviewer #1: The authors conducted a study aiming to assess cellular density and cellular clustering for myeloid cell markers in 129 regions of interest from 55 samples from 35 patients with metastatic ccRCC. CD68+ density was not associated with OS, while high tumor/CD68+ cell clustering was associated with significantly worse OS. I have some concerns on the study design and statistical analysis.

1. The authors need to provide the sample size calculation and power analysis to justify the design of the study.
This study was conceived as a pilot feasibility study to explore the effect of TAM/tumor spatial clustering on overall survival, a relationship that has not been previously assessed. As such, our study design was not based on an explicit statistical hypothesis but we were interested in discovering large effect sizes. A power/sample size analysis using a power of 0.80, and alpha of 0.05, with an 80% event rate indicates that the 35-patient cohort in our analysis would be adequate to detect a minimum detectable hazard ratio (MDHR) of 1.7 for a standardized continuous variable in a univariate Cox proportional hazards regression analysis. This has been included in the methods, results, and discussion sections of the manuscript (lines 143 to 144, and 181 to 184).

We consider this MDHR to be appropriate, as we were interested in discovering large effect sizes. We certainly acknowledge that with a small cohort we are at risk of making Type-II errors in outcomes which were not reported as significant in the manuscript. This limitation has been added to the discussion section of the manuscript (lines 223 to 227).

2. The definition of overall survival is starting from the sample collection time which may varies across patients. The starting time is suggested to use either diagnosis time or treatment starting time.

We explicitly chose OS from the time of sample collection when the study was designed. The specific variables of interest in this study were immune cell clustering and density, which are identified in a snapshot that occurs exactly at the time of sample collection. As such, this reflects survival from the moment in time that these variables were measured, which we feel is the appropriate timeframe.

Additionally, all samples included in this study were collected in the brief pre-treatment window from patients with metastatic ccRCC, and thus the time from diagnosis to sample collection is very short. The median time from diagnosis to sample collection was 26 days (IQR 9 – 42).

3. Have authors conducted assessment to test the Cox proportional hazards assumption?

We have included an assessment of the Cox proportional hazards assumption of the primary Cox regression model (Figure 3C). None of the individual covariates or the Schoenfeld’s global chi-square test violated the proportional hazards assumption. This is included at the bottom of this file as an Appendix.

4. The major limitation of the study is that, there is no validation cohort of the study, it needs to be discussed in the manuscript.

We agree completely. This analysis certainly warrants prospective validation in a larger cohort. We have added this into the limitations section (lines 227 to 228).

Reviewer #2: In this manuscript by Chakriyan et. al., the authors have performed spatial clustering analysis of macrophage markers in metastatic clear cell renal cell carcinoma samples using multiplex immunohistochemistry. The authors have analyzed spatial clusters of macrophage markers with tumor and stromal sections of the tumor tissue to predict the effect on overall survival. It is an interesting approach to analyze and predict overall survival in patients. As mentioned by authors the main limitation of the study is small sample size. The authors show that CD68 spatial distribution has a role to play in predicting OS. The CD163 and CD206 are M2 macrophage markers and are also associated with worse OS in multiple cancer studies. The experimental data would have been more informative and relevant if public database-based OS were compared with the multiplex immunohistochemistry-based spatial clustering of markers. Overall, the article presents an interesting approach to predict overall OS based on spatial distribution of markers. The authors should address the concerns listed:

1. The authors should discuss the relevance of CD163 and CD206 marker clustering in the stromal regions. Since tumor cells secrete a variety of factors that polarize macrophages to M2 type, it is intriguing to note why M2 markers are localized in stromal compartment.
We agree that this is a highly interesting finding. We do not have an answer as to why CD163+ and CD206+ TAMs preferentially cluster into the stromal compartment. However, it is worth noting that this relationship was remarked upon in a prior study, though it was not the purpose of that study and was not quantified (Reference #24 in the manuscript). Further work regarding macrophage chemotaxis into the tumor microenvironment is warranted. For our part, we will first pursue validation of these findings in a larger IF cohort, to confirm this association.

2. Have the authors checked the spatial distribution of CD80 and CD86? Is the staining pattern distribution of these markers similar to CD68?

This is an interesting question, but unfortunately the myeloid panel used in this study did not include the CD80 or CD86 markers.

3. In the Figure 2, authors have stained the tissues for PD-L1 also. The authors should describe the PD-L1 expression in results.

Thank you for noticing this discrepancy. We have included the PDL1 distribution results in the results section (lines 168 to 170).

4. Did the authors observe any difference in spatial distribution of CD68 in tissues when segregated based on gender and does it have any effect on OS also?

These are interesting questions. We have included this data below as an Appendix to this file. There was no difference in CD68/Tumor clustering or OS between genders in our cohort.

**APPENDIX:**

**1.** Testing of the Cox proportional hazards assumption for the survival analysis.

**A.** Schoenfeld’s global chi-square tests for time-dependence of hazard ratios for the individual covariates.

```
               chisq df     p
Age          0.00434  1 0.947
IMDC         2.96618  2 0.283
CD68_Density 0.14951  1 0.699
T_CD68_K50   0.03679  1 0.848
GLOBAL       5.72865  5 0.334
```

**B.** Plots of the scaled Schoenfeld residuals for each individual covariate in the multivariable Cox PH model.

**2.** CD68+ TAM clustering and OS outcomes, stratified by patient gender.

**A.** CD68+/Tumor cellular clustering using nK(25), stratified by gender. Wilcoxon rank-sum test p-value = 0.80.

**B.** KM curves for OS stratified by patient gender (24 males, 11 females). Log-rank test p-value = 0.63.
